# Targeting the Gut–Eye Axis: An Emerging Strategy to Face Ocular Diseases

**DOI:** 10.3390/ijms241713338

**Published:** 2023-08-28

**Authors:** Lucrezia Irene Maria Campagnoli, Angelica Varesi, Annalisa Barbieri, Nicoletta Marchesi, Alessia Pascale

**Affiliations:** 1 Department of Drug Sciences, Unit of Pharmacology, University of Pavia, 27100 Pavia, Italy; annalisa.barbieri@unipv.it (A.B.); nicoletta.marchesi@unipv.it (N.M.); 2 Department of Biology and Biotechnology, University of Pavia, 27100 Pavia, Italy; angelica.varesi01@universitadipavia.it

**Keywords:** ocular microbiota, gut–eye axis, gut microbiota, age-related macular degeneration, uveitis, diabetic retinopathy, dry eye disease, glaucoma, probiotics, symbiotics

## Abstract

The human microbiota refers to a large variety of microorganisms (bacteria, viruses, and fungi) that live in different human body sites, including the gut, oral cavity, skin, and eyes. In particular, the presence of an ocular surface microbiota with a crucial role in maintaining ocular surface homeostasis by preventing colonization from pathogen species has been recently demonstrated. Moreover, recent studies underline a potential association between gut microbiota (GM) and ocular health. In this respect, some evidence supports the existence of a gut–eye axis involved in the pathogenesis of several ocular diseases, including age-related macular degeneration, uveitis, diabetic retinopathy, dry eye, and glaucoma. Therefore, understanding the link between the GM and these ocular disorders might be useful for the development of new therapeutic approaches, such as probiotics, prebiotics, symbiotics, or faecal microbiota transplantation through which the GM could be modulated, thus allowing better management of these diseases.

## 1. Introduction

**Figure 1:**
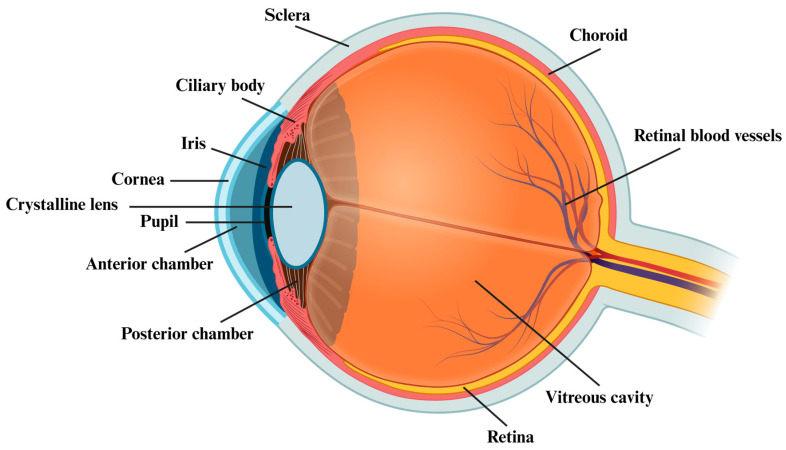
Anatomy of the human eye. Figure created with BioRender.com (accessed on 27 July 2023) (license to S.C.).

The Human Microbiome Project initially revealed the presence of different microbial species (bacteria, viruses, fungi, and archaea) in five distinct districts of the body, including the gastrointestinal and the urogenital tracts, the skin, and the oral and the nasal mucosa, with central roles in health and disease [1,2]. Besides these areas, in recent years, growing interest and research investigations have been focused on the eye and its related ocular surface microbiota (OSM). While the internal compartment of the eye is a sterile environment due to its “closed” anatomical structure (Figure 1) and is protected by an extremely efficient physiological blood–retina barrier (that prevents cells and molecules from moving in and out of the eye as well as the invasion of pathogens), the external ocular surface is constantly exposed to environmental factors, including microorganisms, allergens, and toxins [3,4]. In this regard, the eye developed some defensive frontlines to limit infections and diseases [5]. For instance, the OSM, which comprises a large variety of bacteria belonging to the *Firmicutes* (i.e., *Staphylococcus* and *Streptococcus*), *Actinobacteria* (i.e., *Corynebacterium*), and *Proteobacteria* (i.e., *Acinetobacter* and *Pseudomonas*) phyla, represents a kind of protection against eye infections and diseases. In fact, the OSM is involved in the development of the local immune system, regulation of the host metabolism, and defence against pathogen invasion [4]. However, the existence of a stable and unique OSM is still under debate, since inherent and acquired factors may influence its composition [6]. In this regard, an unbalanced ocular microbiota may lead to pathogenic microbial overgrowth and local or systemic eye inflammation [7]. In the last few years, evidence has suggested the influence of the gut microbiota (GM), composed of six phyla *Firmicutes*, *Bacteroidetes*, *Actinobacteria*, *Proteobacteria*, *Fusobacteria*, and *Verrucomicrobia*, (being *Firmicutes* and *Bacteroidetes* the main ones), on the incidence and progression of eye diseases [1,8]. Indeed, in this respect, many studies have highlighted the existence of a gut–eye axis, where gut microbes can alter the immunity of the eye [9]. Accordingly, alterations in the gut microbial composition may be associated with different ocular diseases, including age-related macular degeneration, uveitis, diabetic retinopathy, dry eye syndrome, and glaucoma [8]. This review will focus on the potential relationship between the GM and ocular health. Specifically, we explore the possible existence of a gut–eye axis and the conceivable role of GM dysbiosis in the development or progression of the ocular diseases mentioned above. Moreover, the present manuscript will also examine the possibility for a therapeutic intervention, such as the potential employment of probiotics, prebiotics, and symbiotics, as well as of faecal microbiota transplantation, to re-establish a “healthier” microbiota, thus helping to prevent or manage these diseases.

## 2. Methods

To review the role of GM and OSM in eye diseases, we carried out an extensive research within the following public databases: PubMed (U.S. National Library of Medicine), Google Scholar, and ClinicalTrials.gov. To focus our search on the selected topics, the following keywords were used alone or in combination: “gut microbiota”, “microbiome”, “ocular surface microbiota”, “inflammation”, “dysbiosis”, “leaky gut”, “gut–eye axis”, “gut permeability”, “probiotic/s”, “prebiotic/s”, “symbiotic/s”, “faecal microbiota transplantation”, “age-related macular degeneration”, “uveitis”, “diabetic retinopathy”, “dry eye”, and “glaucoma”. The resulting literature was analysed and included in our narrative review. Most recent preclinical and clinical studies were preferred, but neither species specificity nor a fixed period of time were imposed on our search.

## 3. The Ocular Surface Microbiota

Throughout evolution, various microorganisms, especially bacteria, colonized the conjunctiva and the cornea as commensals, constituting the so-called OSM [10]. Intriguingly, some studies revealed the capability of the eye to live in complete equilibrium with this community of bacteria. Indeed, the epithelial cells (corneal and conjunctival) of the ocular surface, although constantly in contact with resident commensal bacteria (such as *Staphylococcus epidermidis* or *Propionibacterium acnes*) and their products, do not trigger any inflammatory response against them; to the contrary, when these cells recognize ocular pathogenic bacteria (such as *Pseudomonas aeruginosa*), they produce pro-inflammatory cytokines, including interleukin 1α (IL-1α), tumour necrosis factor α (TNFα), IL-6 and IL-8, chemokines, and interferons. This mode of action suggests the presence of an innate immune system able to distinguish through specific receptors called “pattern recognition receptors”, the commensal from the pathogen-associated molecular patterns, termed, respectively, “microbe-associated molecular patterns” and “pathogen-associated molecular patterns” [11].

### 3.1. OSM Composition

Even though the ocular surface is directly exposed to the same external environment, evidence supports the existence of a unique OSM compared to those related of the facial skin and oral mucosa [12]. Nevertheless, the presence of a stable “core ocular surface microbiota” has been extensively debated and is still under discussion. In the past, the OSM’s composition has been characterized using culture-based methods. In this regard, by culturing swabs of healthy conjunctiva, the most commonly found commensal Gram-positive bacteria, although present in low amounts, were *Staphylococcus*, *Corynebacterium*, *Streptococcus*, and *Propionibacterium* [13]. Furthermore, less frequently, Gram-negative bacteria, including *Haemophilus*, *Neisseria*, and *Pseudomonas* genera, and fungi were also identified [13,14]. However, traditional culture methods are nowadays rarely used due to their inability to detect the complete OSM since these techniques cannot identify slow-growing bacteria as well as uncultivable species [15,16,17]. In this respect, in recent years, the sequencing of bacterial 16S rRNA has been employed to overcome these limitations and to obtain an accurate and complete composition of the healthy human OSM. In 2007, Graham et al. performed the first high-efficiency study in which they identified the bacterial genera present in the conjunctiva samples from 57 healthy individuals using both traditional culture methods and 16S rRNA sequencing [18]. As expected, they found more genera employing the molecular analysis (coagulase-negative *Staphylococcus* sp., *Staphylococcus epidermidis*, *Rhodococcus erythropolis*, *Corynebacterium* sp., *Klebsiella* sp., *Propionibacterium*, *Bacillus* sp., and *Erwinia* sp.) with respect to the culture procedure (only the coagulase-negative *Staphylococcus* and *Bacillus* sp.) [18]. However, the composition of this “core” microbiota has been later disputed and other groups proposed a different putative commensal OSM. For instance, Dong et al., by investigating the OSM from the conjunctival swab of four subjects, discovered 12 genera (*Pseudomonas*, *Propionibacterium*, *Bradyrhizobium*, *Corynebacterium*, *Acinetobacter*, *Brevundimonas*, *Staphylococci*, *Aquabacterium*, *Sphingomonas*, *Streptococcus*, *Streptophyta*, and *Methylobacterium*) regularly present in all the examined samples, which may constitute the presumed conjunctival core microbiota. Specifically, *Proteobacteria* (64%), *Actinobacteria* (19.6%), and *Firmicutes* (3.9%) were found to be the most abundant phyla [17]. Another group classified the bacteria from 105 individuals’ healthy conjunctivae [19], confirming the three previously reported predominant phyla (*Actinobacteria* (46%), *Proteobacteria* (24%), and *Firmicutes* (22%)) and six different genera (*Corynebacterium*, *Streptococcus*, *Propionibacterium*, *Bacillus*, *Staphylococcus*, and *Ralsontia*). In another study, Huang et al., analysing 31 conjunctival samples from healthy subjects, corroborated the existence of the three aforementioned phyla as the most abundant (*Proteobacteria* (46%), *Actinobacteria* (33.9%), and *Firmicutes* (15.5%)) and also identified ten different genera (*Corynebacteria*, *Pseudomonas*, *Staphylococcus*, *Acinetobacter*, *Streptococcus*, *Millisia*, *Anaerococcus*, *Finegoldia*, *Simonsiella*, and *Veillonella*), which could form the supposed OSM “core” [20]. A further relevant study was conducted by Doan and co-workers, who explored the ocular conjunctival microbiota of healthy individuals by using three different techniques (bacterial culture, 16S rDNA gene deep sequencing, and biome representational in silico karyotyping). They found that *Corynebacteria*, *Propionibacteria*, and the coagulase-negative *Staphylococci* were the most abundant organisms in these samples [12]. Last, always in search of the putative OSM “core”, two more recent studies examined healthy conjunctival samples. In detail, Li et al. observed the *Pseudomonas*, *Acinetobacter*, *Bacillus*, *Chryseobacterium*, and *Corynebacterium* genera in 54 conjunctival swab samples [21], while Ozkan et al., testing the conjunctiva of 43 healthy individuals, reported again the prevalent presence of the three above mentioned phyla (*Proteobacteria*, *Actinobacteria*, and *Firmicutes*) with the addition of five genera (*Corynebacterium*, *Sphingomonas*, *Streptococcus*, *Acinetobacter*, and *Anaerococcus*) in one or more subjects. Nevertheless, no specific genus was found in all the individuals, suggesting that the ocular surface does not possess a specific microbiota core signature [22]. However, it should be also underscored that the16S rRNA sequencing has some limitations. For instance, it can detect only bacteria at the level of the genus while it cannot evaluate the functional status of the microbiota [23]. In this regard, some research groups, using next-generation sequencing assays, identified not only bacteria but also other microorganisms, including virus and fungi. For example, Doan et al. reported the presence of the Torque teno virus (belonging to the *Anelloviridae* family, mainly transmitted by faecal–oral route and detected in several tissues) on the ocular surface of healthy subjects [12]; furthermore, two fungal phyla (*Basidiomycota* and *Ascomycota*) and five genera (*Malassezeia*, *Rhodotorula*, *Aspergillus*, *Davidiella*, and *Alternaria*) were also isolated from >80% of 45 samples analysed [24]. Moreover, other viruses have been found at the ocular surface of healthy individuals employing the neutralization assay [25].

Overall, even if metagenomic sequencing has revealed the presence/absence of specific microbial species not previously identified by the traditional culture-based methods, to date, the existence of a stable and unique OSM “core” remains still unclear. In fact, although distinct studies used similar sequencing techniques, they obtained different results. These discrepancies could be due to several factors, including sample size, methods of sampling, contaminations from the DNA extraction kit and polymerase chain reaction reagents, and depth of sampling [26,27,28,29]. Indeed, in regard of the latter, it should be also emphasized that the OSM’s composition appears to have a vertical stratification: for instance, by swabbing the ocular surface with a light pressure, opportunistic and environmental species, such as *Rothia*, *Herbaspirillum*, *Leptothrichia*, and *Rhizobium*, located in the superficial layer, as well as *Firmicutes* (*Staphylococci*) and *Actinobacteria* (*Cornyebacteriae*), placed in the mucosal layer, can be isolated. Meanwhile, *Proteobacteria* (*Bradyrhizobium*, *Delftia*, and *Sphingomonas*) situated in the conjunctival epithelium require deep swabbing [17]. As a consequence, an accurate analysis of the OSM requires (i) a thorough and deep sampling across the different ocular layers; (ii) a combination of culture-based methods and conventional 16S rRNA techniques; and (iii) the use of alternative approaches, such as transcriptional assays, which can measure the activity of the whole microbiota and identify organisms also according to their species or strain [4,17,30,31].

### 3.2. Factors Influencing the OSM Composition

The ocular surface is a dynamic ecosystem and the normal composition of its microorganisms might be hypothetically influenced by different inherent factors, including age, sex, ethnicity, and geographic location, as well as by acquired factors, such as the use of contact lenses, ophthalmic antibiotics, and eye drops [6,32].

Regarding age, some studies reported OSM changes from birth to adulthood [19,33,34,35,36]. For instance, one of the first studies performed by Isenberg et al. underscored qualitative differences between conjunctival microbes of infants born by vaginal delivery and via caesarean section. In detail, the first group of infants had an OSM similar to the one described in the vagina (*Lactobacillus*, *Bifidobacterium*, *Diphtheroids*, *E. coli*, *S. epidermidis*, and *Bacteroides*); instead, the second group showed bacteria resembling the normal skin flora (*Corynebacterium*, *Propionibacterium*, *Diphtheroids*, and *S. epidermidis*) [34]. Moreover, another study reported that the coagulase-negative *Staphylococcus* and the anaerobe *Propionibacterium* represent the most abundant organisms found in infants regardless of the mode of delivery [36]. Interestingly, it has been observed that already two days after birth the OSM starts to change, where *S. epidermidis*, *E. coli*, and *S. aureus* are the most frequent bacteria [35] and continues to evolve over time. Indeed, some groups found significant differences in the bacterial composition of people at different ages [19,33]. For instance, Cavuoto et al., analysing samples from children (<18 years old) and adults, showed a higher abundance of bacteria both at the phylum level (*Proteobacteria*, *Firmicutes*, *Bacteroidetes*, *Fusobacteria*) and at the genus level (*Streptococcus*, *Staphylococcus*, and *Brachybacterium*) in paediatric samples as compared to adults; moreover, they found that the phylum *Actinobacteria* and the genera *Corynebacterium*, *Paracoccus,* and *Propionibacterium* are more abundant in adults [33]. This evidence is similar to the results found by Zhou et al. when comparing children (<10 years old) and individuals > 10 years old. Indeed, they reported a higher richness and Shannon diversity index (used to measure the species diversity) in the first group as compared to the second one [19]. However, other studies reported contrasting results [22,37]. For instance, Ozkan et al. found no effect of age on OSM composition; instead, Wen et al., analysing the OSM of individuals aged between 28 and 84 years, reported a higher Shannon index in the elderly group [22,37].

Regarding sex, although some studies did not report OSM differences between males and females [19,33], others described a sex association. In respect of the latter, Shin et al. found a higher abundance of *Acinetobacter* and members of *Enterobacteriaceae*, as well as a decreased amount of *Anaerococcus,* in females [38]; Ozkan observed a higher Shannon diversity index in males as compared to females but no difference in richness [22]. Moreover, Wen reported differences at the genus level, with a significant decrease in *Propionibacterium acnes* and *S. epidermidis* in males with respect to females and an increase of *E. coli* in females [37]. Last, in the context of ethnicity and geographic location, the OSM composition does not seem to be influenced by these two factors [19].

In terms of acquired factors, the use of contact lenses, ophthalmic antibiotics, and eye drops has been associated with an altered OSM. For instance, one study reported a higher abundance, at the level of the skin below the eye, of opportunistic pathogens, including *Pseudomonas*, *Acinetobacter*, *Lactobacillus*, and *Methylobacterium*, and a lower amount of typical ocular surface genera, such as *Staphylococcus* and *Corynebacterium*, in the OSM of contact lens wearers as compared to the non-wearer ones [38]. Furthermore, another study underscored slight microbial variability between orthokeratology lens wearers and no-lens wearers, as well as between soft contact lens wearers and no-lens wearers. In this respect, the first group (orthokeratology lens wearers) had less abundance of *Bacillus*, *Tatumella,* and *Lactobacillus* as compared to the group not using lens; instead, the soft lens wearers group showed less *Delftia* and more *Elizabethkingia* abundance with respect to no-lens wearers [39]. As expected, the use of ophthalmic antibiotics may also negatively impact OSM. In this regard, Dave et al. reported a significant change in OSM composition after treatment with azithromycin and fluoroquinolonic antibiotics. In detail, they treated 6 patients with azithromycin and 18 patients with fluoroquinolonic antibiotics finding that in azithromycin-treated individuals the amount of the Gram-positive *S. epidermidis* and *S. aureus* was, at baseline, 54.5% and 18.2%, respectively, and 90.9% and 4.5% after antibiotic exposure; in fluoroquinolone-treated patients, the percentages of these bacteria were, at baseline, 45.7% and 6.5%, respectively, with an increase to 63.4% and 13% after intravitreal antibiotic injection. Moreover, treatment with fluoroquinolonic antibiotics decreased the amount of Gram-negative species from 8.7% at baseline to 1.6% [40]. Other studies described the effects of fluoroquinolone (levofloxacin and moxifloxacin) treatment on OSM. After levofloxacin topical use, Ono et al. found a reduction in ocular bacterial diversity, while after ocular moxifloxacin administration, Celebi et al. reported a decrease in the coagulase-negative *staphylococci* (10% vs. 50%), *S. aureus* (5% vs. 20%), and *Corynebacterium* (5% vs. 15%) in the treated group as compared to the control one [41,42]. Regarding moxifloxacin treatment, another clinical study conducted on contact lens wearers found a reduction in Gram-positive commensal bacteria and no change in the amount of Gram-negative bacteria after antibiotic administration [43]. Last, another acquired factor that might influence OSM composition is eye drop use. In this regard, one study described the effect of the eye drops employed to treat glaucoma on conjunctival bacteria, reporting a lower ocular culture-positive rate of bacteria in the treated group as compared to the control one and the presence of Gram-negative bacteria only in the eye drop users [44]. Similar findings were also reported in patients treated with eye drops for curing dry eye syndrome [45].

## 4. Gut–Eye Axis and the Impact on Eye Diseases

**Figure 2:**
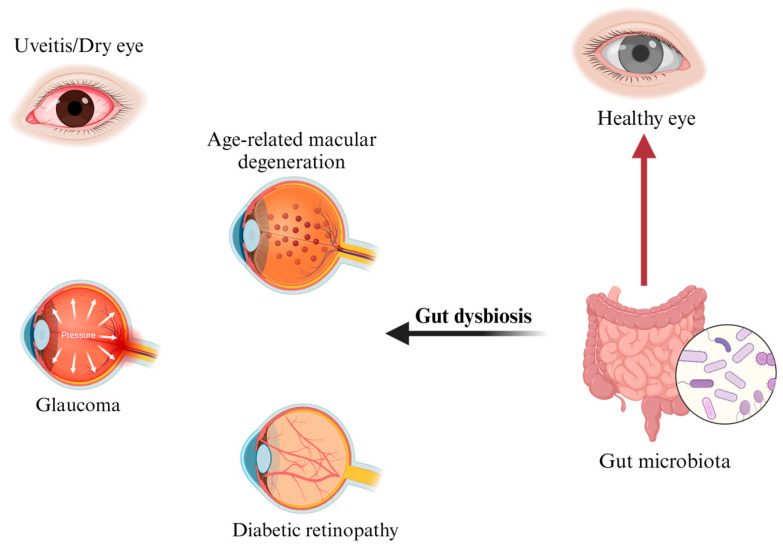
Gut–eye axis and related main ocular diseases. Figure created with BioRender.com (accessed on 27 July 2023) (license to S.C.).

An emerging body of literature is demonstrating that GM plays a key role in the pathogenesis and progression of several extraintestinal disorders. Among these, ocular diseases are known to arise as part of a mutual interplay between the eye and the gut, also known as the gut–eye axis [46] (Figure 2). Within this relationship, gut homeostasis helps maintain retinal health by regulating the host immune system and producing a plethora of anti-inflammatory factors, including short-chain fatty acids (SCFAs), bacteriocins, secondary bile acids, indoles, and polyamines [47]. In dysbiotic conditions, the overgrowth of pro-inflammatory bacteria at the expense of the anti-inflammatory ones promotes gut barrier disruption, metabolic endotoxemia, systemic inflammation, and retinal damage [48]. Although the causal relationship between gut microbiota alterations and eye disease remains to be fully elucidated, it has been hypothesized that a microbial dysbiosis (imbalance) occurring at the ocular surface may trigger an inflammatory response, which could contribute to optic nerve damage and eye disease progression. Age-related macular degeneration, uveitis, diabetic retinopathy, dry eye, and glaucoma have all been associated with gut dysbiosis [49,50,51,52,53,54,55], and their mutual interaction with the intestinal microbiota is analysed below in this review.

### 4.1. Age-Related Macular Degeneration

Age-related macular degeneration (AMD), the leading cause of irreversible blindness in industrialized countries, is characterized by damage to the macula, the central area of the retina, resulting in progressive central vision lost. In the early stages of the pathology, lipids are deposited under the retina, between the retinal pigment epithelium and the so-called Bruch’s membrane, while only at a later time the drusen (the yellow deposits under the retina) are noticeable, thus representing the first visible clinical sign of AMD [56]. Although AMD is considered a progressive retinal degeneration, it is also often associated with chronic intraocular inflammation. There are two forms of AMD depending on the type of macular damage: “dry AMD” and “neovascular AMD” (also called “wet AMD”) [57], where a more severe vision loss is typically associated with the wet form. Several studies have revealed that genetic variants in the complement system together with environmental factors, such as smoking, diet, obesity, or exposure to sunlight, can be involved in the onset of the pathology. Furthermore, since GM has emerged as a key regulator of aging and age-related diseases [58], the relationship between intestinal dysbiosis and the onset of AMD is currently under investigation, given that the number of the available clinical studies is still limited [59]. In this context, there is evidence that mice in which choroidal neovascularization has been induced (a model of AMD) display a significant shift in the GM’s composition as compared to their healthy counterparts, with an increased relative abundance of *Candidatus saccharimonas* at the expense of the genus *Prevotellaceae* NK3B31, which is associated with SCFA production [60]. In addition, it has been reported that the risk of developing AMD is increased in mice fed with a high glycaemic diet, while a low glycaemic food regimen is protective [59,61,62]. Although the underlying mechanisms are not fully understood, high glycaemic conditions are known to induce dysbiosis by favouring the growth of *Clostridiales* at the expense of *Bacteroidales* [62]. Indeed, pro-inflammatory bacteria and their components([i.e., lipopolysaccharides (LPS)), along with a damaged intestinal epithelial barrier, contribute to the establishment of a chronic low grade inflammatory *status* characterized by the expression of TNF-α, IL-6, IL-1β, and vascular endothelial growth factor (VEGF), which favour AMD onset [61]. In contrast, low glycaemic conditions reduce the levels of long-chain polyunsaturated lipids and their peroxidation, increase the abundance of serotonin and C3 carnitine (which protects against retinal damage), and inhibit the production of advanced glycation end products (AGEs; which cause oxidative stress and activate inflammatory pathways), thus preventing AMD development [62]. Nevertheless, in humans, data remain unclear. The intestinal metagenomes of AMD patients and healthy controls were investigated and a different GM composition between the two groups was found. For example, while Burri et al. reported an increase in *Negativicutes* and a decrease in *Oscillibacter* in 57 AMD patients as compared to 58 healthy controls, Zhang et al. showed that AMD subjects are enriched in the pro-inflammatory *Escherichia-Shigella* and depleted in the SCFA-producing *Blautia* and *Anaerostipes* [63,64]. Moreover, Xue et al. identified *Ruminococcus callidus* and *Lactobacillus gasseri* (both belonging to the *Firmicutes* phylum) as the most significantly associated with AMD after combining data from two population cohorts (Chinese and Swiss) [65], while Zinkernagel et al. showed an increase in *Oscillibacter, Anaerotruncus, Eubacterium ventriosum*, and *Ruminococcus torques* in AMD patients versus control subjects that, instead, presented a higher content of *Bacteroides eggerthii* [49]. Still, a reduced *Firmicutes/Bacteroides* ratio seems to characterize AMD across the different studies [63,65]. Metabolically, glutamate degradation, arginine synthesis, and L-alanine fermentation are upregulated in the intestinal microbes from AMD patients [49]. Of note, both glutamate excitotoxicity and glutamate deficiency are associated with retinal dysfunction [66,67], and alanine is found dysregulated in retinopathies [68]. In contrast, AMD-associated GM shows a decrease in transcripts encoding for the fatty acid elongation pathway, which is known to play a crucial role in retinal integrity [49,69]. Overall, despite what these data have underlined, i.e., an association between AMD and intestinal dysbiosis, further investigations on the interplay among GM, GM-derived metabolites, and AMD are mandatory; moreover, managing GM composition with alternative strategies could be useful for lowering the risk of progression at the early stages of the disease.

### 4.2. Uveitis

Uveitis is one of the leading causes of blindness in the world and is most commonly diagnosed in individuals between 20 and 60 years old. The disease can be classified, based on its anatomical location within the eye, in anterior uveitis, intermediate uveitis, posterior uveitis, and panuveitis (inflammation involving all the ocular parts) or, depending on its etiology, in infectious and non-infectious uveitis [70,71,72]. The pathogenesis of uveitis, as well as the underlying mechanisms through which the condition develops, is complex and multi-factorial. A major cause of the disease is an immune dysregulation leading to autoinflammation and/or autoimmunity [73]. Other key factors involved in the pathogenesis are infections, such as viral, bacterial, or parasitic infections, which can directly invade the eye or induce an immune response that leads to inflammation. In addition, in susceptible individuals, genetic and environmental factors may also contribute to the development of the disease [74,75]. Emerging evidence suggests a potential association between uveitis and alterations in the GM composition or function [76,77]. One of the first pieces of evidence on the relationship between GM and the pathogenesis of uveitis came in 2014, when Lin et al. reported that transgenic rats harbouring the human HLA-B27 gene (a well-known risk factor for acute anterior uveitis) had decreased relative abundance of caecal *Bacteroides vulgatus* and *Rikenellaceae* in favour of *Prevotella* species [78]. Furthermore, mice with experimental autoimmune uveitis treated with oral antibiotics showed a reduced abundance of *Dorea*, *Lactobacillus*, *Clostridium*, and *Coprococcus*, which are associated with disease symptoms [79,80]. Conversely, the administration of oral antibiotics clinically ineffective for this disease failed to reverse the GM composition of affected mice, demonstrating the importance of the intestine in achieving therapeutic success [79,80]. In line with this evidence, GM reshaping has been reported to mediate the clinical benefits associated with the intake of antimetabolites or anti-inflammatory substances in preclinical uveitis models [81,82]. This is the case for the antibiotic minocycline, which is known to prevent retinal infiltration by pro-inflammatory immune cells [83]. Besides this conventional mechanism of action, minocycline treatment modulates the GM composition towards the overgrowth of *Streptococcus hyointestinalis*, *Ruminococcus bromii*, and *Desulfovibrio*. This in turn stimulates the GM production of histamine, pantothenic acid, and propionic acid, which possess immunomodulatory and anti-inflammatory activities [83]. Other GM-derived metabolites involved in counteracting disease pathogenesis are SCFAs and bile acids. Indeed, it has been reported that SCFAs injected intraperitoneally can cross the blood–eye barrier and penetrate the eye, where they attenuate ocular inflammation [84]. Moreover, given that decreased levels of secondary bile acids have been detected in serum and faeces of experimentally induced uveitis mice [85], a re-establishment of optimal bile acid levels is sufficient to attenuate disease symptoms and reduce nuclear factor-kappa B (NF-kB) activation in dendritic cells mainly by modulating TGR5 (the bile acid membrane receptor) signalling [85].

Until now, only a few studies have investigated a link between GM and uveitis in humans. In humans, acute anterior uveitis patients show a trend towards increased *Veionella, Prevotella,* and *Streptococcus* (pathogenic and pro-inflammatory bacteria) at the expense of the protective *Roseburia, Faecalibacterium, Ruminococcus,* and *Lachnospira* as compared to matched healthy controls [51,86]. This bacterial profile results in higher levels of the pro-inflammatory metabolites azelaic acid and linoleic acid, which have been found linked to uveitis, oxidative stress, and T cell dysregulation [86,87]. Other studies have been conducted on subjects with Behçet’s syndrome, a blood vessel disorder associated with eye inflammation. The subgroup of patients affected by this syndrome, and also showing uveitis, is characterized by depleted *Blautia, Coprococcus, Dorea*, and *Lachnospiraceae* in favour of increased *Succinivibrionaceae, Bilophila,* and *Stenotrophomonas* [88,89]. Of note, differences in various taxa have been observed across different disease phenotypes, with *Lachnospiraceae* characterizing Behçet’s syndrome patients with uveitis [90,91]. Moreover, transplantation of faecal matter from Behçet’s syndrome patients with ocular inflammation to experimental autoimmune uveitis mice resulted in disease exacerbation, intestinal epithelial barrier damage (as measured by increased LPS in the circulation), increased expression of the pro-inflammatory cytokines IL-17 and interferon-gamma (IFN-γ), and decreased abundance of the SCFAs butyric, valeric, and propionic acids [92,93]. Even though these studies emphasize the role of the microbiota in uveitis, further research is needed to better characterize the patterns of dysbiosis in these patients. Nevertheless, several mechanisms have been already proposed regarding the involvement of the microbiota in the pathogenesis of uveitis, including antigenic mimicry, dysbiosis leading to impaired microbiota-dependent immune homeostasis, and migration of gut mucosa-associated lymphocytes towards peripheral sites. Notably, antigen mimicry is the process by which self-reactive T cells are produced as a result of the cross-reactivity of gut microbial peptides with self-antigens [94]. Although the specific microbial antigens triggering uveitis through mimicry have not yet been identified, there are some promising studies concerning other autoimmune diseases that could help to explain the pathogenesis of uveitis [95,96].

Overall, these studies highlight a potential week contribution of GM in the regulation of disease pathogenesis and therapy response; however, a clear intestinal signature associated with the disease remains to be established. Moreover, understanding the link between uveitis and GM might have implications for the development of novel therapeutic approaches.

### 4.3. Diabetic Retinopathy

Diabetes mellitus (DM), a metabolic disorder characterized by disturbances in glucose metabolism by insulin deficit/resistance and chronic hyperglycaemia, is a significant threat to global health. The prevalence of DM has increased rapidly over the past few decades and is estimated to rise from 463 million in 2019 to 700 million in 2045 worldwide [97]. The most common eye complication in DM patients is diabetic retinopathy (DR), which is the leading cause of blindness worldwide [98].

It is known that DM patients (with prediabetes, type 1, and type 2 diabetes) are associated with GM dysbiosis [99,100]; however, besides playing an important role in the pathogenesis of type 2 diabetes mellitus [101], GM is emerging as a key player in diabetes complications, including diabetic retinopathy (DR) [102]. For example, while obesity-associated GM aggravates the DR phenotype [103], intermittent fasting appears protective [104]. Moreover, evidence from gene-wide association studies reports that the *Peptococcaceae* and *Christensenellaceae* families are protective, while the genera *Aldecruzia*, *Eubacterium rectale* group, and *Ruminococcaceae* UCG-011 increase the risk of developing DR [105]. When compared to healthy controls, DR patients show an increased relative abundance of *Faecalibacterium, Lachnospira, Alistipes*, and *Roseburia* at the expense of *Blautia, Akkermansia*, and *Anaerostipes* [106,107]. This bacterial setting reflects alterations at the level of the faecal metabolomic profile, with a marked reduction in nicotinic acid, carnosine, succinate, and niacinamide found among DR patients as compared to controls [107]. Of note, mitochondrial function, inflammatory regulation, and antioxidant defences greatly rely on these metabolites [108,109]. GM alterations were also observed when comparing diabetic patients with and without DR. In the latter case, decreased levels of *Veionella* and *Bacillus* were reported in favour of an increase in *Prevotella* [107], which has a pro-inflammatory potential [110]. Additional faecal metagenomic studies and meta-analyses confirmed a reduction in the SCFA-producing *Bifidobacterium, Faecalibacterium*, and *Lactobacillus*, counterbalanced by an increase in *Bacteroidetes*, *Proteobacteria*, and *Burkholderiaceae*, which are considered pro-inflammatory [102,111,112]. Among the faecal metabolites, α-linolenic acid and arginine–proline metabolisms are the most dysregulated in DR patients. In this respect, increased levels of the pro-oxidant traumatic acid (a monounsaturated dicarboxylic acid naturally occurring in plants) are observed at the expense of armillaramide (a sphingosine ceramide), which is implicated in membrane stability and lipid metabolism [107].

However, there is still much debate about whether the correlation between GM dysbiosis and the disease is inherent to the diabetic pathology or whether it is a side effect due to the medications used to treat the disease. In regard of the latter, several studies have found that diabetic medications, particularly metformin, influence the GM, and medications have indeed been identified as a confounding variable in microbiota studies on diabetic individuals [113]. Thus, future studies aimed at evaluating intestinal microbial dysbiosis in diabetic patients must be stratified to isolate confounding variables such as drugs. Moreover, overall, these data underline that different GM compositions are associated with different diabetes stages and complications. In this respect, the possibility of using GM-based signatures that are able to stratify DR patients has been suggested [54] and should be further investigated.

Furthermore, it should be mentioned that DM patients are not only characterized by changes in GM composition, but also by alterations in ocular surface features. In this respect, tear film dysfunction, increased conjunctival metaplasia, decreased conjunctival goblet cell density, sub-basal nerve density, corneal sensitivity, and delayed epithelial and stromal wound healing have been described [114,115]. Specifically, these changes were associated with type 2 DM and were shown to be proportional to the severity of DR. Moreover, cases of infection such as conjunctivitis, corneal ulcer, and endophthalmitis were also reported in these patients, thus mirroring an alteration in the ocular protective immune response together with a decrease in cytokine production and an impairment in cellular immune response functions [116,117,118,119,120,121,122,123,124,125,126]. All together these alterations may disturb the normal ocular surface microenvironment and the microbiota itself, thus possibly having an impact on disease development/progression. In addition, considering that in the advanced stage of DR the common treatments are intravitreal drug injections or intraocular surgery, these procedures may allow the ocular surface microbes to enter into the eye, thus contributing to intraocular infections [123,127], as supported by the presence of Gram-negative bacteria on the ocular surface of DM with respect to non-DM subjects [128,129,130,131,132].

Concerning the OSM profile, one study performed by Ham et al. revealed differences between diabetic patients with DR and healthy subjects [133]. In detail, at the phylum level, *Proteobacteria* were found to be more abundant in the diabetic group (82% vs. 56.1%), while *Firmicutes* (17.1% vs. 4.7%), *Cyanobacteria* (8.7% vs. 2.1%), *Bacteroidetes* (6.8% vs. 3.7%), and *Actinobacteria* (10.4% vs. 6.9%) were more prevalent in the control individuals. Moreover, they found variations also at the genus and family level: in particular, at the genus level, *Acinetobacter* (43.36% vs. 2.96%), *Sphingomonas* (4.49% vs. 2.99%), and *Ralstonia* (3.71% vs. 3.70%) appeared to be overrepresented in the diabetic patients when compared to control subjects, where the most frequent bacteria are *Corynebacterium* (6.51% vs. 2.38%) and *Pseudomonas* (4.80% vs. 1.67%). Meanwhile, at the family level, *Bradyrhizobiaceae* (16.15% vs. 3.33%) and *Neisseriaceae* (3.72% vs. 1.36%) are most frequent in the control group when compared to diabetic patients [133]. Another important study showed differences in the OSM, on one hand, between DM and non-DM groups and, on the other hand, between DM groups with proliferative DR (PDR)/non-proliferative DR (NPDR) and without DR [134]. At the phylum level, *Proteobacteria* were highly prevalent in both non-DM and DM groups (40.56% vs. 42.18%), followed by *Firmicutes* (32.43% vs. 34.83%), *Actinobacteria* (13.43% vs. 10.71%), and *Bacteroidetes* (9.35% vs. 6.71%). At the class level, *Alphaproteobacteria* were abundant in the non-DM group, while *Gammaproteobacteria* in the DM group; moreover, at the family level, *Enterobacteriaceae* were more frequent in the diabetic group than in the non-DM group, while *Neisseriaceae* were prevalent in the diabetic patients with DR as compared to DM without DR and non-DM groups. Last, at the genus level, *Escherichia-Shigella* were significantly abundant in the DM patients as compared to the non-DM group, while *Pseudomonas* was more abundant in diabetic patients with PDR (6.05%) as compared to the other groups (DM-NPDR (2.35%), DM without DR (2.42%), and non-DM group (3.05%)) [134]. Concerning the conjunctival flora, in a study in 53 type 2 diabetics, Adam et al., found a higher frequency of Gram-negative bacteria, including *E. coli* and *Klebsiella*, on the ocular surface as compared with 43 healthy controls. Rates for bacterial isolations were determined as 38.5% in diabetic patients and 34.9% in non-diabetic controls. *Staphylococcus aureus* was isolated in 30% of cases in the DM group, while 20% tested positive for *E. coli*, 10% for coagulase-negative *Staphylococcus*, 10% for *Klebsiella pneumoniae*, and 30% for multiple bacteria. In the non-diabetic group, 53.3% of patients were positive for *Staphylococcus aureus*, while coagulase-negative *Staphylococcus* was isolated in 26.7%, *Klebsiella pneumoniae* in 6.7%, and multiple bacteria in 13.3% of patients. Although there was no statistically significant difference in the number of isolated bacteria between the diabetic and non-diabetic groups, Gram-negative bacterial colonization was significantly higher in DM patients [135].

In relation to type 1 DM, findings have been reported in a diabetic animal model. Specifically, type 1 diabetes was induced in rats via administration of streptozotocin, a diabetogenic agent that selectively destroys pancreatic beta cells. In this study, the conjunctival flora demonstrated a reduced diversity compared with control rats injected with saline. This was accompanied by a reduction in *Staphylococcus*, *Aerococcus*, and *Klebsiella* and by an increase in *Enterococci*, *Kocuria*, *Enterobacter*, and *Proteus* at the ocular surface. These findings are in line with the concept that species such as *Staphylococcus* are normally present in healthy conjunctiva, while *Enterococci* are known to cause infection [136]. Although it is known that DR may take many years to develop [137], these data suggest that a change in bacterial composition at the ocular surface may take part in the pathogenesis of DR itself. Of note, it is important to emphasize that, similarly to the gut, there is no unique signature of OSM dysbiosis at the basis of all the different ocular diseases.

### 4.4. Dry Eye Disease

Dry eye disease (DED) is an ocular surface disorder with a high prevalence worldwide, characterized by a loss of tear film homeostasis, resulting in excessive evaporation of tears, hyperosmolarity, inflammation, and neurosensory abnormalities. Diabetes mellitus is one of the risk factors and other possible causes include abnormal enzyme metabolism and decreased mucin secretion [138]. Within this context, an important role is played by Meibomian glands, located in the eyelids, which are responsible for secreting oily components to the tear film, thus protecting the ocular surface from dryness, discomfort, or damage. Indeed, Meibomian gland dysfunction often leads to evaporative dry eye syndrome. In addition, changes in the normal microbiota and oxidative metabolism contribute to DED by inducing inflammation at the ocular surface. In fact, a pathological alteration in the microbial composition of the ocular surface can induce an immune response and the production of reactive oxygen species, which in turn activate the inflammasome by increasing tear film instability and osmolarity [139]. Nevertheless, the literature does not clearly define which microbial species undergo changes in DED and Meibomian gland dysfunction. However, although there are no conclusive results, it has been suggested that a specific resident bacterium of the ocular surface, namely the *Corynebacterium*, is probably associated with this disease [4].

It has long been established that Sjögren’s syndrome, an immune disorder characterized by dry eye signs, often associates with GM alterations [140]. Indeed, there is evidence that the colonization of germ-free mice with the GM derived from Sjögren’s syndrome patients decreases the abundance of CD4^+^ forkhead box P3 (FOXP3)^+^ Treg cells in cervical lymph nodes and promotes corneal barrier damage following dry eye induction [141]. Moreover, feeding IL-14α transgenic mice (overexpressing human IL-14α; a model of Sjögren’s syndrome) with a high-fat diet correlates with reduced GM richness, overgrowth of intestinal *Deferribacterota*, and worse dry eye manifestations [142]. Still, in another mice study in which dry eye pathology was induced by desiccation stress (applied to the ocular surface using a ventilator) with or without scopolamine (an antimuscarinic agent), the obtained findings show that this type of stress is able to change the GM, with an increase in the *Proteobacteria* and a decrease in *Bacteroides* and *Firmicutes* in comparison with unstressed mice [143]. Reduced GM diversity has also been reported in humans, where a depletion of *Faecalibacterium*, *Bacteroides*, and *Parabacteroides* and an overgrowth of *Streptococcus*, *Blautia*, and *Escherichia/Shighella* have been observed in Sjögren’s syndrome patients as compared to healthy controls [144]. In these patients, dry eye signs are particularly associated with an increased relative abundance of the phyla *Proteobacteria, Bacteroidetes*, and *Actinobacteria* at the expense of *Firmicutes* [145]. Moreover, independent studies reported the importance of *Prevotella* in regulating tear secretion and dry eye severity [55,146].

When considering Sjögren’s syndrome-independent dry eye manifestations, preclinical studies show that an overall shift in β diversity correlates with disease symptoms [147]. Differentially abundant microbes include *Proteobacteria, Prevotella, Helicobacter*, and *Alistipes*, which are all positively correlated with disease severity [147]. Of note, antibiotic-treated dry eye mice show worsened disease symptoms and reduced goblet cell (a source of mucin for tears and of different types of mucins for the ocular surface) density through decreased abundance of *Clostridium* and stimulated growth of pro-inflammatory and pathogenic gut microbes (i.e., *Enterobacter, Pseudomonas,* and *Escherichia/Shigella*) [144]. Concerning GM-related metabolites, restoring the levels of SCFAs may represent a promising therapeutic strategy [148,149]. Indeed, it has been reported that SCFA transporters, such as SLC5A8, are expressed in the epithelia of the conjunctiva and cornea and that butyrate administration reduces the type I interferon response and ocular inflammation in mice [149]. Furthermore, the intake of lactoferrin (a protein contained in tears) dampens the expression of pro-inflammatory cytokines via an increase in the abundance of SCFA-producing bacteria [148]. Overall, despite these promising preclinical results, clinical evidence remains limited to Sjögren’s syndrome. Thus, further studies are needed to better clarify the direct effect of GM alterations on dry eye symptoms [150].

Concerning the ocular microbiota, studies performed in animals have shown that two types of CD4^+^T lymphocytes with opposing immunological roles, T helper cells type 17 (Th17) and regulatory T cells (Treg), interact with the resident microbiota of the eye and have been related to dry eye syndrome. Through such immunological interactions, the resident ocular microbiota may potentially trigger and perpetuate the inflammation occurring in dry eye syndrome. Indeed, Th17 cells are pro-inflammatory and produce cytokines, including IL-17, which can activate an immune response against pathogenic microorganisms. Conversely, Treg behave as anti-inflammatory cells under normal conditions and play a role in immune tolerance. The proper ratio of these two lymphocyte subsets ensures the correct modulation of the immune function, while an imbalanced ratio is associated with the onset of various inflammatory diseases and autoimmunity. Of interest, resident gut microbes and their metabolites can affect this ratio. Based on this evidence, further studies involving patients with moderate to severe dry eye syndrome analysed the tears of individuals upon awakening and characterized the microbiota of tears collected from closed eyes (on awakening from sleep). The results highlight that the tears collected from closed eyes of patients affected by dry eye syndrome harbour a distinctly different and more enriched microbiota than the one observed in healthy subjects. Specifically, the significant differences in terms of the relative abundance were observed in the genera, including *Bacteroidetes*, *Pseudomonas*, and *Meiothermus*, suggesting that a greater microbial diversity within the human eye represents a feature of dry eye syndrome [151]. Furthermore, another study investigated the OSM of patients with DM and DED. In detail, individuals were divided into four groups: healthy subjects, patients with DED only, with DM only, and diabetic individuals with DED (DM with DED group) [138]. At the phylum level, the DM with the DED group showed lower levels of *Proteobacteria* as compared to the DED-only group (44.90% vs. 55.37%), higher levels of *Firmicutes* as compared to the control group (31.84% vs. 30.39%), differences in the *Actinobacteria* amount as compared to the DED-only group and the control one (9.31% vs. 5.44%; 9.31% vs. 8.77%, respectively), and higher levels of *Bacteroidetes* as compared to the other three groups (4.55% vs. 1.73% (controls), 2.60% (DED-only), 2.39% (DM-only)); at the genera levels, the DM with the DED group showed lower relative abundance of *Corynebacterium* and higher relative abundance of *Ochrobactrum*, *Bacillus*, *Cupriavidus*, and *Lactococcus* as compared to the DM-only group and the DED-only group. No statistically significant differences were found concerning the other comparisons [138].

### 4.5. Glaucoma

Glaucoma is a disabling neurodegenerative disorder and the second leading cause of blindness worldwide. The retinal ganglion cells (RGCs), which play a crucial role in the development of this disease, undergo death or dysfunction, and their axons lose the intrinsic capacity to regenerate [152,153,154]. The progressive damage not only involves the RGCs themselves, but also the optic nerve. However, current treatments mainly target the intraocular pressure (IOP), the most prominent risk factor for glaucoma, and a cure has yet to be discovered [155]. Nevertheless, although some patients maintain relatively stable visual fields after their IOP is lowered to a target level, the majority of them, the so-called progressors, continue to lose vision despite having an IOP that is seemingly well controlled upon medical therapy [156]. Hence, protecting RGCs from deterioration/degeneration and favouring axon regeneration after damage, together with a reinstatement of the proper nerve connections, can constitute supportive therapeutic approaches aimed to enhance visual restoration, thus making more effective the traditional pharmacologic intervention [157,158,159]. In this regard, there is an urgent need for novel neuroprotective/neuroenhancing therapies along with the identification of biomarkers for an early diagnosis of glaucoma, especially in the progressors who are likely to benefit from more effective and/or non-IOP-related therapies.

An autoimmune component has long been suspected in glaucoma pathology [160]. A peripheral inflammation, such as that linked to high oral bacterial loads, has also been reported to be associated with an increased incidence of glaucoma [161]. Chen and co-workers in 2018 published an article demonstrating that the transient elevation of IOP induces two phases of neural damage in glaucomatous mice: (i) an acute phase correlated with IOP elevation; (ii) a prolonged phase observed after the IOP returns to the normal range. The authors document that an elevated IOP stimulates CD4^+^T cells to enter the retina and that these T cells are responsible for the prolonged phase of neurodegeneration in glaucoma [162]. This interesting study also demonstrates that CD4^+^T cells are “trained” by the commensal microflora to recognize bacterial heat shock proteins (HSPs) and then to cross-react with host (mouse or human) HSPs. Remarkably, in two different mouse models of glaucoma, the animals raised in the absence of the commensal microflora (germ-free mice) were devoid of HSP-reactive T cells, and they did not develop optic nerve damage in the setting of elevated IOP [162]. This study provides convincing evidence that, in glaucoma, a commensal microflora-mediated immune mechanism underlies the progressive death of neurons and that T cells are pre-sensitized by the symbiotic microbiota. Also, although dysregulations of the intestinal microbiota have been deeply characterized in various neurodegenerative conditions, little is known about the relationship between gut dysbiosis and glaucoma [52]. There is evidence that the risk of developing glaucoma is higher in patients with chronic irritable bowel syndrome than in their unaffected counterparts, suggesting the importance of the gut–eye axis in disease pathogenesis [163]. When looking into the GM composition, preclinical evidence reports a shift towards increased *Verrucomicrobia, Akkermansia*, and *Bacteroides* in glaucoma rats as compared to controls, which is accompanied by reduced levels of the antioxidant glutathione in blood circulation [52]. In humans, the faecal microbiota of patients with glaucoma is enriched in *Enterobacteriaceae, Prevotellaceae*, and *Escherichia coli* and depleted in *Megamonas*, *Blautia*, and *Fusicatenibacter* when compared to controls, although no significant differences in overall bacterial diversity were observed between the two populations [53,164]. However, it should be stressed that some discrepancies remain in the determination of the GM-dependent metabolic profile between glaucoma patients and controls. In this respect, while in one study an enrichment in isocitrate and citric acid have been reported to correlate with the disease, reduced levels of citric acid have been shown in another study dealing with an independent cohort [53,164]. Moreover, butyrate, a gut bacterial metabolite, has been shown to lower IOP independently of blood pressure changes in rats [165], while an increased level of trimethylamine (an uremic toxin produced by GM) has been observed in the aqueous humour of patients with glaucoma [166]. Indeed, there is emerging evidence suggesting a potential link between the intraocular microbiota and the development of the disease. In order to define the intraocular microbiota in the context of the glaucoma, Deng and co-workers conducted a preliminary comparison between the results obtained with metagenomic methods from one group of 41 otherwise normal eyes undergoing cataract surgery and those obtained from an additional 38 patients who had a diagnosis of AMD or glaucoma at the time of cataract surgery. The bacterial community in the aqueous humour from all the three patient cohorts showed a classical individuality similar to that found in other body districts. Interestingly, the alpha diversities of the intraocular microbial communities were significantly different among these three types of patients, despite all patients having bacteria as the major component of their intraocular microbiota [167]. Concerning OSM, some studies summarized in Petrillo’s work in 2020 have reported an increased abundance of certain bacteria, such as *Streptococcus*, *Staphylococcus*, and *Corynebacterium*, in the ocular surface of glaucoma patients as compared to healthy controls [32]. In this regard, Chang in 2022 [168] studied the ocular surface composition from swabs collected from both eyes of 17 subjects: 10 patients with asymmetric/unilateral glaucoma undergoing a topical glaucoma therapy in only one eye and 7 age-matched healthy controls with no history of ocular disease or eyedrop use. Comparisons among patients’ glaucomatous eye treated with eyedrops, patients’ contralateral eye without eyedrops, and healthy control eyes were made for microbial diversity and composition [168]. In agreement with the studies mentioned above, the samples obtained from the patients’ treated and untreated eyes showed both a significantly greater alpha-diversity and relative abundance of Gram-negative organisms as compared to healthy controls. These differences in OSM composition were associated with decreased tear film measures and distinct protein synthesis pathways, thus suggesting a potential link between microbial alterations and ocular surface inflammation. However, despite all these findings suggesting a potential association between OSM and glaucoma, the exact mechanisms by which the ocular microbiota may influence the development and progression of glaucoma are still not fully understood. Therefore, further research is needed to establish a causal relationship and to determine the specific roles of the different microorganisms in glaucoma pathogenesis. Furthermore, more studies are needed to identify possible GM metabolites specific for glaucoma patients.

## 5. Therapeutic Strategies

### 5.1. Probiotics

Probiotics are living microorganisms that provide beneficial effects to the host if consumed in an adequate amount [169,170]. To date, several anti-inflammatory commensal microbes have been considered possible probiotics, with strains belonging to *Streptococcus* [171], *Enterococcus* [172], *Bacillus* [173], *Pediococcus* [174], *Escherichia coli* [175], and *Leuconostoc* [176]. However, the commonly used probiotic formulations usually contain *Lactobacilli* and *Bifidobacteria,* which are often altered in several gastrointestinal and extraintestinal disorders [177,178,179,180]. Reported direct or indirect benefits associated with probiotic consumption include reduced gastrointestinal inflammation and pro-inflammatory cytokine secretion, inhibition of pathogen growth, improvement of epithelial barrier integrity, and increased SCFA production [181,182,183,184]. Mainly, the anti-inflammatory effect of probiotics arises from the modulation of the JAK/STAT and NF-kB pathways in the gut’s epithelial cells, promoting tissue healing and improving cellular response to stress [185]. Moreover, probiotics influence the activity of Th1, Th2, Th17, dendritic cells, NK cells, B lymphocytes, and macrophages through the modulation of toll-like receptor signalling [186,187]. By interacting with the mucosal immune system, probiotics induce the production of IgA, bacteriocins, and defensins, which are then secreted into the lumen and reinforce host immunity [188]. Based on these advantages, probiotics have been considered a potential treatment for various diseases associated with GM dysbiosis, including intestinal bowel syndrome, ulcerative colitis, rheumatoid arthritis, major depressive disorder, and even cancer [189,190,191,192]. Although in the context of eye disorders the evidence remains scarce, results from preclinical studies are promising, and preliminary data coming from human studies are encouraging the design of further clinical trials [193,194,195,196] (Table 1) [197,198,199,200,201,202,203,204,205,206,207,208,209,210,211,212,213]. There is evidence that mice with ocular inflammation receiving eye drops containing the cell-free supernatant of the probiotic *Lactiplantibacillus plantarum* show reduced ocular inflammation, lower amount of infiltrating inflammatory cells, and diminished levels of the pro-inflammatory cytokines TNF-α and IFN-γ [205]. These benefits appear to be mediated by the anti-inflammatory action of *Lactiplantibacillus plantarum* supernatant in retinal pigment epithelial cells, as inflammatory and oxidative stress markers (i.e., IL-6, IL-8, thiobarbituric acid (used to assess lipid peroxidation), and nitric oxide (NO)) are reduced after probiotic treatment [205]. In line with the anti-inflammatory action of probiotics, mice models of autoimmune uveitis receiving *Escherichia coli* O83:K24:H31 before symptom manifestation showed reduced T cell immunoreactivity, lower NO production from macrophages, and increased intestinal secretion of antimicrobial peptides, thus blocking disease onset and progression [200]. Of note, no benefits were observed when *Escherichia coli* Nissle 1917 was administered following the same experimental protocol, highlighting the importance of strain selection for a therapeutic success [200]. In humans, data obtained from a case report conducted on a 21-year-old woman affected by refractory acute anterior uveitis showed that combining steroids with probiotics improves visual acuity and limits ocular inflammation [207]. While in this study a probiotic mixture composed of *Bifidobacterium bifidum*, *Bifidobacterium lactis*, and *Bifidobacterium breve* was used [207], the potential benefits of using other probiotic formulations as therapeutic treatment for uveitis remains to be investigated.

Additional evidence on the immunomodulatory potential of probiotics comes from two independent prospective comparative pilot studies carried out in 20 adults and 26 children with chalaziosis. In both cohorts, adding a probiotic mixture composed of *Streptococcus thermophilus*, *Lactobacillus delbrueckii*, and *Lactococcus lactis* to the standard of care shortened the time required to heal the disease [202,203]. In light of these evidence, larger clinical trials are warranted.

Dry eye is another ocular disease leading to eye inflammation for which probiotics have been considered [214]. In this respect, IRT5 (a probiotic mixture composed of *Lactobacillus reuteri*, *Lactobacillus acidophilus*, *Lactobacillus casei*, *Streptococcus termophilus*, and *Bifidobacterium bifidum*) intake proved effective in restoring tear secretion in NOD.B10.H2b mice (an established autoimmune dry eye model) [199,204]. This improvement was accompanied by an increased abundance of T regulatory cells at the expense of immunoreactive CD8^+^IFN-γ^+^ T cells [204]. At the intestinal level, reshaping of the GM towards an increased relative abundance of *Lactobacillus hamster* and *Lactobacillus helveticus* may mediate the IRT5 anti-inflammatory effect [199]. When tested in a preventive setting, IRT5 administration still proved efficient in increasing tear production, but no improvements in ocular inflammatory markers suggest that more studies are needed to establish the optimal intervention protocol as well as to uncover the mechanism of action [206].

Besides IRT5, promising results were obtained upon oral gavage with *Bifidobacterium bifidum* and *Lactiplantibacillus plantarum* to mice after dry eye induction [213]. In addition to enhanced tear production, this treatment was associated with increased goblet cell density and improved ocular inflammatory parameters (as measured by reduced levels of TNF-α, IL-1β, and myeloperoxidase in favour of higher IL-10 expression) [213]. GM diversity in probiotic-treated mice resembled the controls, with increased content of *Muribaculaceae* and *Lactobacillaceae* at the family level at the expense of the phyla *Actinobacteria* and *Verrucomicrobia* [213]. However, human studies are required to validate these preclinical findings.

The link between dysregulations in the renin–angiotensin system and diabetic microvascular complications is well established [215]. In this context, it has been reported that the loss of the vasoprotective proteins of the renin–angiotensin system, such as ACE2 (angiotensin-converting enzyme 2) and Ang-(1-7) are associated with disease onset and exacerbation [216,217]. This aspect, together with the fact that gut dysbiosis in DR is often characterized by a decreased relative abundance of anti-inflammatory microbes, including *Faecalibacterium, Lachnospira, Blautia, Streptococcus, Bifidobacterium,* and *Lactobacillus* [218,219], have led to the engineering of common probiotics to replenish ACE2 and Ang-(1-7) levels. Indeed, an engineered strain of *Lactobacillus paracasei* expressing the human ACE2 protein, besides ameliorating hyperglycaemia, is able to reduce systemic inflammation, improve the integrity of the gut epithelial barrier (as measured by the markers zonula occludens-1 (ZO-1) and p120-catenin), and counteract the development and progression of DR when administered preventively or therapeutically in *Akita* and endothelial nitric oxide synthase (eNOS)^−/−^ mice (mouse models of diabetes) [209,212]. At the eye level, reduced content of pro-inflammatory cytokines in the retina, decreased abundance of acellular capillaries, and prevention of retinal ganglion cell loss contribute to reverse the disease phenotype [209,212]. These benefits are accompanied by improved blood–retinal barrier function (as measured by increased ZO-1 and decreased vascular cell adhesion molecule 1 (VCAM-1) levels) and higher expression of ABCG1 in the retinal pigment epithelium [208]. Of note, ABCG1 is a protein involved in the transport of lipids from the blood into the retina, a process that is dysregulated in DR [220]. Similarly to ACE2, the possibility of engineering *Lactobacillus paracasei* to make it capable of secreting Ang-(1-7) has been investigated [211]. eNOS^−/−^ mice receiving this engineered probiotic 3 times a week for 8 weeks showed significant improvements in ocular inflammatory markers (as measured by reduced levels of monocyte chemoattractant protein 1 (MCP-1), IL-1β, and TNF-α), a consistent limited loss of vascular capillaries in the retina, and a reduced abundance of retinal ionized calcium binding adaptor molecule 1 (Iba1^+^) microglial cells (which are generally increased in DR) [217,221].

Other eye diseases that may benefit from the anti-inflammatory and antioxidant effects of probiotics are glaucoma and AMD. In glaucoma, the intake of a fermented maize emulsion rich in lactic acid bacteria may attenuate the clinical signs of the disease by reducing the intraocular pressure, preventing the loss of ganglion cells, and protecting against gliosis [222]. Of note, these benefits are accompanied by increased levels of brain-derived neurotrophic factor (BDNF), which has a protective role against retinal damage [222]. Concerning oxidative stress, results obtained from a randomized controlled trial involving 57 patients with AMD showed that an 8-week intake of a probiotic mixture containing species from *Bacillus, Lactobacillus*, and *Bifidobacterium* lowers the levels of the pro-oxidant factor malondialdehyde (MDA) while systemically increasing the total antioxidant capacity [201]. However, the absence of a clear clinical effect associated with the antioxidant improvements may suggest that longer treatment periods are required to achieve a therapeutic benefit.

Overall, the results from the preclinical studies are promising. However, the insufficient amount of clinical evidence highlights the need for large cohort randomized controlled trials to validate the preventive and therapeutic role of probiotics in the context of eye diseases [195]. Besides the gut–eye axis, studies investigating the direct effect of probiotics on the modulation of ocular surface microbiota are also required. To date, only one randomized controlled trial has been reported linking the efficacy of probiotics to the composition of the ocular surface microbiota [223]. In this study, 60 individuals were randomized to receive eye drops alone or combined with a probiotic mixture composed of *Enterococcus faecium* and *Saccharomyces boulardii*. Although the improvement in dry eye symptoms among the probiotic group has been associated with a trend towards a re-establishment of OSM homeostasis, the direct role of an in situ probiotic administration on disease amelioration remains to be fully clarified [223]. Future studies should determine the feasibility of targeting eye bacteria as an alternative and additive strategy to the more common GM modulation.

### 5.2. Prebiotics and Symbiotics

Prebiotics are undigestible carbohydrate substances rich in fibres that act as food for the GM, with beneficial outcomes for the host [224]. Examples of prebiotics include, but are not limited to, galacto-oligosaccharides, fructans, inulin, glucose-derived oligosaccharides, and starch [225]. Given their ability to selectively favour the growth of beneficial anti-inflammatory bacteria over their anti-inflammatory counterpart, prebiotics have been considered a preventive or therapeutic treatment for various pathologies, including Crohn’s disease [226], skin allergies [227], cardiovascular diseases [228], and neurological disorders [229,230]. To date, however, only few studies have investigated the potential of prebiotics in the context of eye disorders. Concerning the OSM’s composition, innovative contact lens formulations capable of releasing the prebiotic resveratrol in the tear fluid have been proposed. Preliminary evidence indicates that the use of these lenses is associated with a reduced growth of harmful biofilm-producing bacteria, such as *Pseudomonas aeruginosa* and *Staphylococcus aureus*, decreased inflammatory parameters, and improved antioxidant potential [231]. Moreover, it has been claimed that the beneficial effects of hyaluronan (an extracellular matrix mucopolysaccharide often found in eye drops) are largely mediated by its prebiotic activity [232]. Accordingly, mice treated with hyaluronan show increased SCFA-producing bacteria, decreased abundance of harmful sulphate-reducing bacteria, and improved antioxidant capacity (as measured by higher superoxide dismutase (SOD) and lower MDA levels, respectively) [232]. Taking advantage of these preliminary results, clinical trials directed to test OSM modulators should be evaluated.

Considering the gut–eye axis, there is evidence that a diet favouring the growth of butyrate-producing bacteria may reduce dry eye symptoms in patients with Sjögren’s syndrome [233]. Although the mechanism of action seems to involve the targeting of aquaporin5, which is upregulated in dry eye conditions [234], more data are needed to unravel the complex relationship between GM modulation and symptom improvements.

If the actual benefit of prebiotics alone remains poorly defined in the context of eye disorders, promising clinical data are emerging upon their combination with probiotics (Table 1). To date, the co-administration of probiotics and prebiotics (the so-called symbiotics) proved successful in reducing the clinical symptoms and the inflammatory parameters (i.e., C-reactive protein (CRP), hs-CRP, and erythrocyte estimated sedimentation rate) in an uveitis patient [197]. In this case study, fructo-oligosaccharides were mixed with seven probiotic strains (*Lactobacillus rhamnosus, Lactobacillus bulgaricus, Lactobacillus casei, Lactobacillus acidophilus, Bifidobacterium breve, Bifidobacterium longum,* and *Streptococcus thermophilus*) and administered orally twice a day for 7 months [197]. Similarly, the concurrent intake of a probiotic mixture (containing species from *Lactobacillus, Bifidobacterium*, and *Streptococcus*) and the prebiotic NutriKane D (sugarcane + pectin) increased tear secretion and improved the ocular surface features in a randomized controlled trial conducted in 41 dry eye patients [210]. The simplest formulation, composed of *Bifidobacterium lactis*, *Bifidobacterium bifidum*, and fructo-oligosaccharides, also reduced the dry eye symptoms when tested in 40 patients randomized to receive symbiotics or placebo daily for 1 month [198]. Overall, despite the few studies that have been carried out, positive results have been reported thoroughly. However, it remains to be clarified to what extent prebiotics contribute to the beneficial effects observed upon symbiotic intake, as these may be primarily driven by probiotics alone.

### 5.3. Faecal Microbiota Transplantation

Faecal microbiota transplantation (FMT) consists of the transfer of fresh or frozen faecal material from healthy donors to a patient’s intestine to restore eubiosis [235]. Although delivery through colonoscopy remains the preferred route, its intrinsic invasiveness and the risks associated with the procedure have recently led to the consideration of enema, nasogastric delivery, or oral pills as alternative transplantation techniques [236,237]. To date, FMT has been successfully used for the treatment of refractory or recurrent *Clostridium difficile* infections [238,239] and is now under consideration for primary *Clostridium difficile* infections [240], Crohn’s disease [241], intestinal bowel syndrome [242], chronic fatigue syndrome [243], obesity [244], neurodegenerative diseases [229,230], and neuropsychiatric disorders [245]. Beneficial effects of FMT include re-established eubiosis [246], reduced levels of inflammation [247,248], increased amounts of SCFAs [247], and prevention of gut epithelial barrier leakage [248]. In light of these benefits, emerging studies have investigated the potential of FMT as a modulator of the gut–eye axis, with a particular focus on autoimmune uveitis and Sjögren’s syndrome [194,249]. It has been reported that germ-free mice and antibiotic-treated mice show enhanced corneal barrier disruption, reduced goblet cell density, increased infiltration of lymphocytic cells (particularly CD4^+^ IFN-γ^+^ T cells) in the lacrimal gland, and higher levels of pro-inflammatory cytokines (i.e., IL-12 and IFN-γ), thus resembling the dry eye phenotype observed in Sjögren’s syndrome patients [144,250,251]. This was accompanied by an increased relative abundance of pro-inflammatory *Enterobacteria* belonging to the genera *Enterobacter, Pseudomonas*, and *Escherichia/Shigella* in the stool of antibiotic-treated animals [144]. Of note, FMT from wild-type C57BL/6J mice was sufficient to reduce the CD4^+^ IFN-γ^+^ T cell infiltration and to recover the dry eye phenotype, suggesting the feasibility of this approach in the context of eye-related diseases [250,251]. More recently, Parker et al. reported that stool transfer from old mice into their young counterpart increases intestinal barrier permeability and retinal inflammation (as measured by enhanced levels of the pro-inflammatory cytokines and chemokines CCL11, CXCL11, and IL-1β) and decreases the levels of the retinal epithelial protein RPE65, which plays an important role in vision [252]. Conversely, old mice receiving FMT from young donors showed a reversed phenotype [252]. Concerning humans, clinical data remain limited. Results from an open label non-randomized clinical trial conducted in 10 patients with Sjögren’s syndrome showed that two FMT administrations in a 1-week interval improve dry eye symptoms in 50% of them at a 3-month follow-up, with no reported adverse events [253]. Despite these benefits, no overall changes in the profile of the GM were reported in the long-term, with only the abundance of few bacterial species resembling the donor (i.e., *Euryachaeota, Methanobacteria, Lentisphaeria, Prevotella*, and *Ruminoclostridium*) [253]. Therefore, although preliminary positive results have been reported, more studies are needed to clarify the feasibility of FMT as a possible therapeutic intervention in eye-related disorders. Moreover, emerging evidence suggests that the interplay between GM and intestinal fungal species should be considered to maximize FMT efficacy, as alterations in diversity and richness of gut fungal species were reported in uveitis patients compared to controls [254].

## 6. Conclusions

Although a growing body of evidence suggests the existence of a gut–eye axis and thus the potential involvement of an altered GM composition in ocular disease onset and progression, further studies are required to understand the direct and solid link between GM and the eye together with the underlying molecular mechanisms. Furthermore, understanding the exact microbiota profiles (GM and ocular microbiota) associated with ocular diseases could be the starting point for developing new specific treatments. In this regard, among the various microbiota-modulating approaches to date, probiotics are the most promising in the context of eye disorders. However, the traditional oral administration of probiotics restraints their activity towards the GM without directly influencing the OSM’s composition. In this respect, topical formulations that include probiotics to the standard eye drop treatment are of great interest as they can specifically modulate OSM, but data remain scarce. It is likely that future studies will be performed to evaluate the feasibility and efficacy of this emerging approach. Indeed, the obtained findings will prove essential to determine the direct and indirect (via GM) impact of OSM on eye diseases, as the exact mechanism behind the gut–eye axis is yet to be fully elucidated. Even when considering the gut–eye axis, although significant evidence is emerging from probiotic studies, only a few have investigated the impact of the same probiotic formulation. Indeed, the extensive variability across independent studies remains a major limitation when designing clinical trials. Therefore, studies aimed at defining the best-performing probiotic strain or mixture, the most efficient therapeutic window, and the optimal dosage are urgently needed.

In conclusion, the truth behind the presented associations is the fact that, generally speaking, the microbiota is a crucial modulator of inflammation. Certainly, inflammatory processes play a key role in ocular disease development, thus giving a strong contribution to the observed associations between the microbiota and the described diseases. Therefore, further studies are encouraged to help in defining the importance of the OSM (both direct or indirect via GM) in eye homeostasis and the mechanisms involved in the development of eye pathologies, with the aim of designing and then testing more specific ocular formulations able to influence the microbiota’s composition and, hence, possibly impact the development/progression of eye diseases.

## Figures and Tables

**Table 1:** Preclinical and clinical evidence on the use of probiotics and symbiotics for eye diseases.

PROBIOTICS						
Probiotic	Strain	Condition	Experimental Model	Study Design	Results	Ref.
*Lactiplantibacillus plantarum*	CRL759	Inflammatory eye disorder	Mice	Eye drops of *Lactiplantibacillus plantarum* supernatant	↓ Ocular TNF-α, IFN-γ ↓ Pro-inflammatory cell infiltrate	[205]
*Lactiplantibacillus plantarum + Bifidobacterium bifidum*	NK151 (*L. plantarum*) and NK175 (*B. bifidum*)	Dry eye	Mice, n = 8 Vehicle (n = 4) Probiotics (n = 4)	Daily for 10 days	↑ Tear secretion ↑ Goblet cell density ↑ IL-10 ↓ IL-1β, TNF-α, myeloperoxidase ↓ Gut inflammation ↓ *Verrucomicrobia, Actinobacteria* ↑ *Lactobacillaceae, Muribaculaceae*	[213]
*Streptococcus thermophilus + Lactococcus lactis + Lactobacillus delbrueckii*	ST10 (*S. thermophilus*), and LLC02 (*L. lactis*) and subsp. *bulgaricus* (*L. delbrueckii*)	Chalaziosis	Humans, n = 20: Std (n = 10) Std + probiotics (n = 10)	Daily for 3 months	↓ Time for chalazia resolution	[202]
			Children, n = 26: Std (n = 13) Std + probiotics (n = 13)			[203]
*Escherichia coli*	Nissle 1917	Uveitis	Mice, n = 52 Placebo (n = 25) Probiotics (n = 27)	3 times/week in a preventive or therapeutic setting	↓ T cell immunoreactivity (lymph nodes) ↓ Inflammation (Peyer’s patches) ↓ iNOS (macrophages) ↑ Antimicrobial peptides (gut)	[200]
*Bifidobacterium lactis + Bifidobacterium bifidum + Bifidobacterium breve*	BL04 (*B. lactis*), BB01 (*B bifidum*), and BR03 (*B. breve*)	Uveitis	Human, n = 1 Std + probiotics	Daily for 1 year	↓ Ocular inflammation ↑ Visual acuity	[207]
ACE2-expressing *Lactobacillus paracasei*	N/A	Diabetic retinopathy	Mice, n = 12 Untreated (n = 6) Probiotics (n = 6)	3 times/week for 3 months	↓ Gut lacteal damage expression ↑ Gut epithelial barrier integrity ↑ Gut endothelial barrier integrity ↓ Plasma LDL ↓ Blood–retinal barrier damage ↓ Acellular capillaries (retina)	[208]
			Mice, n = 21 Untreated (n = 9) Probiotics (n = 12)	9 months (prevention group) or 3 months (intervention group)	↓ Diabetic retinopathy ↑ Gut barrier integrity ↓ Gut anti-inflammatory macrophages ↓ IL-2, IL-1β, IL-17, IL-6, TNF-α, IFN-γ (gut)	[209]
	ATCC 27092		Mice, n = 16 Vehicle (n = 8) Probiotics (n = 8)	3 times/week for 8 weeks (eNOS^−/−^ mice) or 12 weeks (*Akita* mice)	↓ Acellular capillaries (retina) ↓ TNF-α, ICAM-1, MCP-1, IL-1α (retina)	[212]
Ang-(1-7)-expressing *Lactobacillus paracasei*	ATCC 27092	Diabetic retinopathy	Mice, n = 12 Vehicle (n = 6) Probiotics (n = 6)	3 times/week for 8 weeks (eNOS^−/−^ mice) or 12 weeks (*Akita* mice)	↑ Vascular capillaries (retina) ↑ Ganglion cells (retina) ↓ MCP-1, TNF-α, IL-1β, VEGF, ICAM-1 (retina)	[211]
Multi-probiotics mixture	*Lactobacillus acidophilus, Lactobacillus casei, Bacillus coagulans, Bifidobacterium bifidum, Bifidobacterium longum,* and *Bifidobacterium lactis*	Age-related macular degeneration	Humans, n = 57 Placebo (n = 29) Probiotics (n = 28)	Daily for 8 weeks	↑ HDL cholesterol ↑ TAC (plasma) ↓ MDA = clinical symptoms	[201]
Multi-probiotics mixture (IRT5)	*Lactobacillus acidophilus, Lactobacillus casei, Lactobacillus reuteri, Bifidobacterium bifidum* and *Streptococcus thermophilus*	Dry eye	Mice, n = 35 Vehicle (n = 16) Probiotics (n = 19)	Daily for 3 weeks	↑ Tear secretion ↓ Ocular staining score ↑ *Lactobacillus hamsteri* and *Lactobacillus helveticus, Christensenellaceae* ↓ CD8^+^ IFN-γ^+^ cells	[199]
		Uveitis and dry eye	Mice, n = 21 Vehicle (n = 10) Probiotics (n = 11)			[204]
		Dry eye	Mice, n = 25 Vehicle (n = 16) Probiotics (n = 9)	Daily for 11–12 days		[206]
*Saccharomyces boulardii* and *Enterococcus faecium*	MUCL 53,837 (*S. boulardii*) and LMG S-28935 (*E. faecium*)	Dry eye	Humans, n = 60 Std (n = 30) Std + probiotics (n = 30)	N/A	↓ Dry eye symptoms	[198]
**SYMBIOTICS**						
**Probiotic**	**Prebiotic**	**Condition**	**Experimental Model**	**Study Design**	**Results**	**Ref.**
*Lactobacillus rhamnosus* + *Lactobacillus bulgaricus* + *Lactobacillus casei* + *Lactobacillus acidophilus* + *Bifidobacterium breve* + *Bifidobacterium longum* + *Streptococcus thermophilus*	FOS	Uveitis	Humans, n = 1 Symbiotics	Twice a day for 7 months	↓ Uveitis ↓ CRP, hs-CRP, ESR	[197]
MULTIBIOTIC^TM^	NutriKane D	Dry eye	Humans, n = 41 Placebo (n = 18) Symbiotics (n = 23)	4 months	↓ Ocular surface disease ↓ Dry eye ↑ Tear secretion	[210]
*Bifidobacterium lactis + Bifidobacterium bifidum*	FOS		Humans, n = 40 Std (n = 20) Std + symbiotics (n = 20)	Daily for 1 month	↓ Dry eye ↑ Intestinal homeostasis	[198]

Abbreviations: ACE2: angiotensin-converting enzyme 2; Ang-(1-7): angiotensin-(1-7); CRP: C-reactive protein; eNOS: endothelial nitric oxide synthase; ESR: erythrocyte estimated sedimentation rate; FOS: fructo-oligosaccharides; HDL: high-density lipoprotein; hs-CRP: high-sensitivity CRP; ICAM-1: intracellular adhesion molecule 1; IFN-γ: interferon gamma; IL: interleukin; iNOS: inducible nitric oxide synthase; LDL: low-density lipoprotein; MCP-1: monocyte chemoattractant protein-1; MDA: malondialdehyde; Std: standard of care; TAC: total antioxidant capacity; TNF-α: tumour necrosis factor alpha; VEGF: vascular endothelial growth factor; ↓: decrease; ↑: increase.

## Data Availability

Not applicable.
